# Right paraduodenal hernia complicated with small bowel obstruction and midgut rotation: a case report

**DOI:** 10.11604/pamj.2026.53.150.52218

**Published:** 2026-04-02

**Authors:** Mohammed Ba Gunaid, Nada Mohsen Salama, Augustin Prochotsky

**Affiliations:** 1 Department of Surgery, Poprad Hospital, Comenius University, Bratislava, Slovakia,; 2 National Cancer Diagnostic and Interventional Radiology Department, Interventional Radiology Unit, Cairo University, Cairo,; 3 Department of Surgery, St. Cyril and Metoda Hospital, Comenius University, Bratislava, Slovakia

**Keywords:** Right paraduodenal hernia, internal hernia, small bowel obstruction, case report

## Abstract

Right paraduodenal hernia is a rare congenital internal hernia resulting from abnormal midgut rotation. Although paraduodenal hernias represent the most common type of congenital internal hernia, the right-sided variant is an uncommon cause of small bowel obstruction and is often challenging to diagnose preoperatively. We report the case of a 46-year-old woman who presented with a 48-hour history of acute abdominal pain, nausea, and vomiting, without any prior history of abdominal surgery. Physical examination revealed tenderness in the epigastric and mesogastric regions without signs of peritonitis, while laboratory findings showed leukocytosis. Plain abdominal radiography demonstrated small bowel air-fluid levels, and contrast-enhanced computed tomography suggested small bowel obstruction secondary to an internal hernia. Urgent exploratory laparotomy revealed a right paraduodenal hernia through Waldeyer´s fossa with viable entrapped small bowel loops. The herniated bowel was successfully reduced, and the mesenteric defect was closed without the need for bowel resection. Right paraduodenal hernia should be considered in patients presenting with small bowel obstruction in the absence of prior abdominal surgery. Early diagnosis using computed tomography and prompt surgical intervention are crucial to prevent strangulation and ischemic complications.

## Introduction

Internal hernias are defined as the protrusion of intra-abdominal viscera through congenital or acquired apertures within the peritoneal cavity. According to autopsy results, the incidence of internal hernias is between 0.2% and 0.9% [1]. Paraduodenal hernias (PDHs) constitute the most frequent subtype of congenital internal hernias, accounting for approximately half of all internal hernia cases, yet they represent only a small fraction of all causes of intestinal obstruction. The two principal forms, left and right PDH, differ in embryologic origin and anatomical features. Right paraduodenal hernia (RPDH) is a rare congenital internal hernia resulting from incomplete rotation of the midgut and abnormal peritoneal fixation during embryologic development. It occurs through Waldeyer´s fossa, a mesenteric defect located to the right of the superior mesenteric vessels. Right PDHs are less common than left PDHs, with a reported approximate ratio of 1:3 [2]. The clinical presentation of RPDH is often nonspecific, ranging from vague abdominal discomfort to acute intestinal obstruction, making preoperative diagnosis challenging. Contrast-enhanced computed tomography (CT) has emerged as the most valuable imaging modality, typically revealing clustered small bowel loops with associated displacement of mesenteric vessels, which can facilitate early diagnosis and prompt surgical management [3]. Early recognition of internal hernias, including RPDH, is essential, as delayed intervention increases the risk of strangulation, ischemia, and adverse outcomes [4]. This case is unique due to the rare occurrence of a right paraduodenal hernia, presenting as acute small bowel obstruction. Preoperative diagnosis by computed tomography allowed timely surgical intervention, and early management enabled successful reduction without bowel resection, preventing ischemic complications.

## Patient and observation

**Patient information:** a 46-year-old woman presented to the emergency department with a 48-hour history of acute abdominal pain accompanied by nausea and vomiting. She denied any history of prior abdominal surgery or chronic gastrointestinal symptoms. She had no known chronic medical conditions and was not taking any regular medications. Additionally, she denied recent trauma, fever, or constitutional symptoms such as weight loss or fatigue. Her past medical history was otherwise unremarkable.

**Clinical findings:** on admission, vital signs were stable: blood pressure 110/70 mmHg, pulse 96 beats per minute, respiratory rate 18 breaths per minute, temperature 36.8°C, and oxygen saturation 96% on room air. Abdominal examination revealed diffuse tenderness in the epigastric and mesogastric regions without guarding or rebound tenderness.

**Timeline of the current episode:** symptoms began 48 hours prior to presentation with progressive abdominal pain, nausea, and vomiting. Imaging and laboratory evaluation were performed shortly after admission, followed by surgical intervention approximately four hours later.

**Figure 1:**
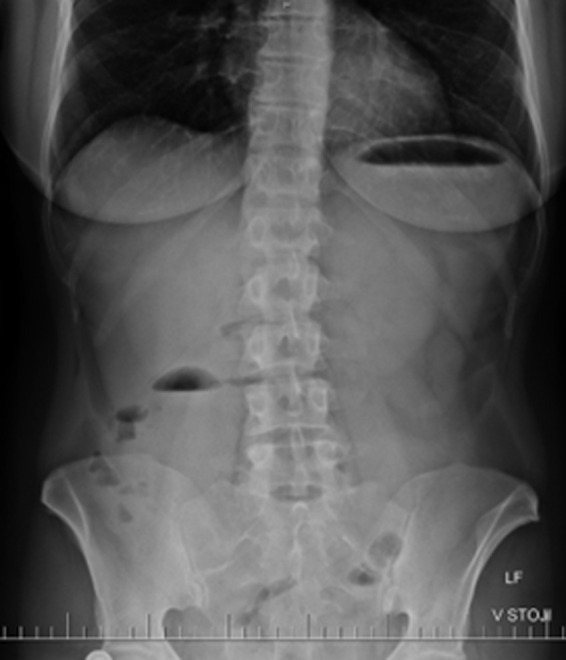
plain abdominal radiography demonstrated small bowel air-fluid levels predominantly localized to the right mesogastric region, without evidence of pneumoperitoneum

**Figure 2:**
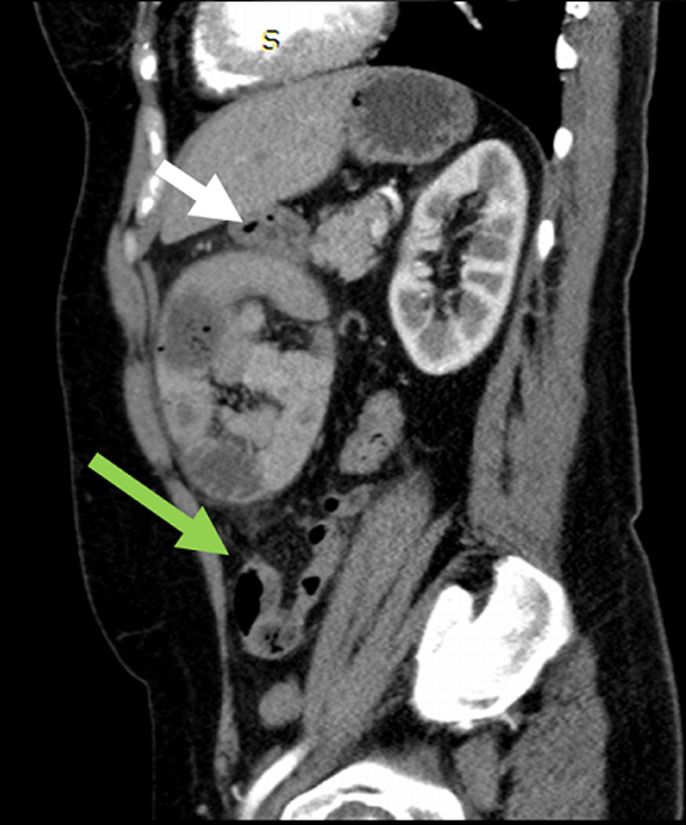
sagittal reformatted image showing the inferiorly displaced transverse colon and hepatic flexure of the colon (green arrow), and the stomach and pylorus are superiorly displaced (white arrow)

**Figure 3:**
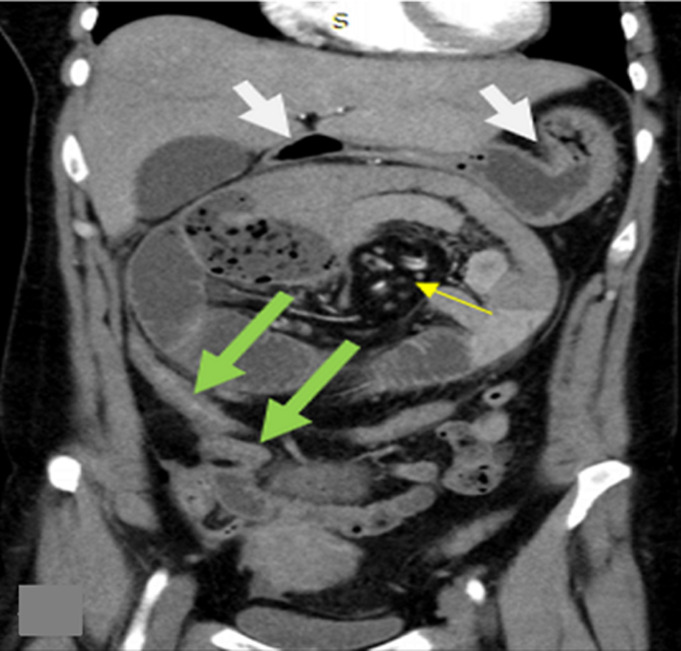
coronal reformatted image showing distended, centrally located jejunal bowel loops with air-fluid levels confirming small bowel obstruction; the stomach and pylorus are superiorly displaced and effaced against the hepatic hilum (white arrow), with inferiorly displaced transverse colon (green arrow), and the whirlpool sign of the small intestinal mesentery indicating midgut rotation (yellow arrow)

**Figure 4:**
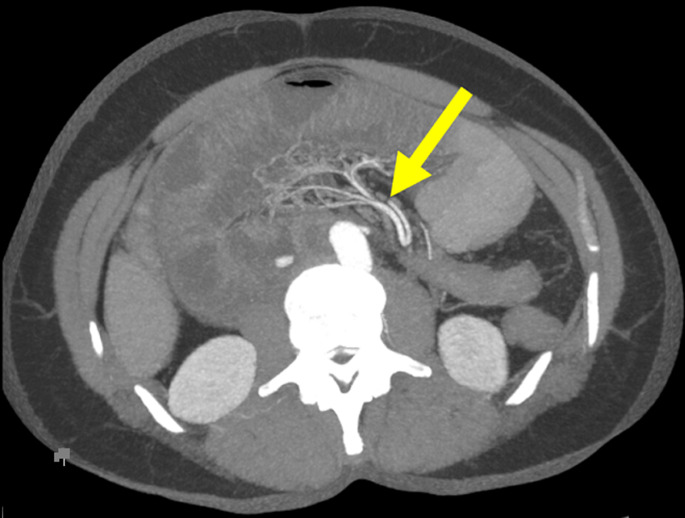
axial maximum intensity projection images showing the whirlpool sign of the small intestinal mesentery with the bowel vasculature rotated 180 degrees to the right (midgut rotation), (yellow arrow)

**Figure 5:**
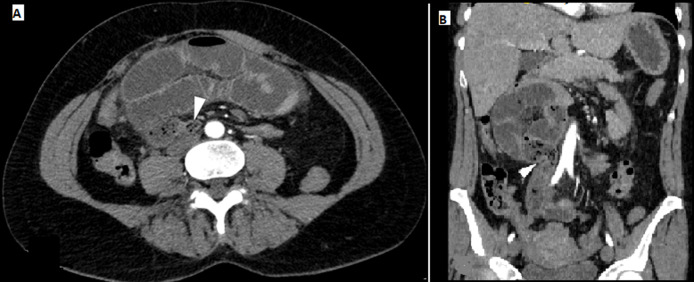
A,B) internal hernia containing jejunal bowel loops just beyond the duodenojejunal junction with transition point noted (white arrowhead) to the right of the spine

**Diagnostic assessment:** i) X-ray: plain abdominal radiography demonstrated small bowel air-fluid levels predominantly in the right mesogastric region, without pneumoperitoneum (Figure 1); ii) computed tomography (CT) scan: contrast-enhanced CT confirmed small bowel obstruction due to an internal hernia, with features suggestive of a right paraduodenal hernia (Figure 2, Figure 3, Figure 4, Figure 5); iii) laboratory tests: laboratory tests showed leukocytosis with a white blood cell (WBC) count of 12,400/mm^3^, while liver and kidney function tests, pancreatic enzymes, ionogram, C-reactive protein (CRP), and coagulation profile were all normal.

**Therapeutic intervention:** urgent exploratory laparotomy was performed approximately four hours after admission. A single prophylactic intravenous dose of cefuroxime (1.5 g) and metronidazole (500 mg) was administered preoperatively. Intraoperatively, a right paraduodenal hernia through Waldeyer´s fossa was identified, with small bowel loops herniating through a mesenteric defect measuring approximately 6 cm. The bowel was reduced and found to be viable, with no ischemia or necrosis. The defect was closed using a continuous non-absorbable suture. A prophylactic appendectomy was also performed due to adhesions involving the appendix.

**Follow-up and outcomes:** the postoperative course was uneventful. The patient resumed oral intake without complications and was discharged on postoperative day three. At the 14-day follow-up, she remained asymptomatic with normal bowel function.

**Patient perspective:** the patient described her symptoms as sudden and distressing, with abdominal pain and vomiting. Following surgery, she reported gradual improvement and expressed satisfaction with the care provided. At her outpatient follow-up on postoperative day 14, she noted continued recovery with regular bowel movements and overall clinical improvement.

**Informed consent:** the patient included in this research gave written informed consent to publish the data contained within this study.

## Discussion

Internal hernias are an uncommon but important cause of small bowel obstruction (SBO), accounting for less than 1% of all cases of intestinal obstruction. Despite their low incidence, they are clinically significant because of the increased risk of strangulation and bowel ischemia if diagnosis and management are delayed [5]. Internal hernias may be congenital or acquired; congenital forms arise from developmental anomalies of midgut rotation and peritoneal fixation, whereas acquired types are more frequently associated with previous surgery, trauma, or inflammatory processes [6]. Among congenital internal hernias, paraduodenal hernias are the most common subtype. They result from abnormal rotation of the midgut and failure of fusion of the mesentery to the posterior parietal peritoneum. Paraduodenal hernias are classified as left- or right-sided depending on their anatomical location. Although left-sided variants are more frequently reported, right paraduodenal hernias remain a well-recognized but rare entity [7]. The herniation occurs through a congenital mesenteric defect, allowing small bowel loops to become entrapped behind the mesocolon, which predisposes to obstruction and possible vascular compromise. Clinically, internal hernias present with variable and often nonspecific symptoms. Patients may report intermittent abdominal pain, nausea, and vomiting, or present acutely with signs of mechanical SBO.

The absence of previous abdominal surgery should raise suspicion for congenital internal hernia in cases of obstruction [6]. Because of the nonspecific clinical presentation, preoperative diagnosis has historically been difficult, and many cases were identified only during laparotomy. The role of imaging, particularly computed tomography (CT), has become central in improving diagnostic accuracy. Blachar *et al*. described characteristic CT findings in internal hernias, including clustering of small bowel loops in an abnormal anatomical location, displacement of mesenteric vessels, and signs of closed-loop obstruction in complicated cases [8]. Recognition of these radiologic features allows earlier diagnosis and reduces the risk of delayed intervention. Early identification is essential because prolonged incarceration may lead to strangulation, bowel necrosis, and increased mortality [5]. Surgical management remains the definitive treatment for paraduodenal hernias. The fundamental operative principles include reduction of the herniated bowel, careful assessment of bowel viability, and correction or closure of the mesenteric defect to prevent recurrence. In cases where ischemia is present, bowel resection may be required. Traditionally, open laparotomy has been the standard approach, particularly in emergency settings [6,7]. In the present case, early recognition of small bowel obstruction in a patient without prior abdominal surgery prompted urgent surgical exploration. The entrapped bowel was viable, and primary closure of the defect was performed successfully without the need for resection. The uneventful postoperative recovery emphasizes the importance of timely diagnosis and intervention. Overall, internal hernias-although rare-should remain in the differential diagnosis of small bowel obstruction, particularly in patients without previous abdominal operations. Awareness of characteristic imaging findings and adherence to prompt surgical management are essential to prevent life-threatening complications.

## Conclusion

Right paraduodenal hernia is a rare but potentially life-threatening cause of small bowel obstruction that should be suspected in patients without prior abdominal surgery. Early recognition through contrast-enhanced CT imaging is crucial to prevent strangulation and bowel ischemia. Prompt surgical reduction and closure of the mesenteric defect remain the cornerstone of management and are associated with excellent outcomes when performed before irreversible bowel compromise occurs.
